# Association of single nucleotide polymorphism rs3803662 with the risk of breast cancer

**DOI:** 10.1038/srep29008

**Published:** 2016-06-28

**Authors:** Yuan Yang, Wenjing Wang, Guiyou Liu, Yingcui Yu, Mingzhi Liao

**Affiliations:** 1 College of Life Sciences, Northwest A&F University, Yangling, Shaanxi, China; 2 School of Life Sciences, Fudan University, Shanghai, China; 3 Research Center for Translation Medicine, East Hospital, Tongji University School of Medicine, Shanghai, China; 4 School of Life Science and Technology, Harbin Institute of Technology, Harbin, Heilongjiang, China; 5 College of Natural Resources and Environment, Northwest A&F University, Yangling, Shaanxi, China

## Abstract

Large scale association studies have identified the single nucleotide polymorphism rs3803662 associated with breast cancer risk. However, the sample size of most studies is too small. Here, we performed this meta-analysis to make the result more convincing. Relevant articles published up to 2016 were identified by searching the PubMed database. 13 studies, involving a total of 29405 participants, were included in the meta-analysis. Odds Ratios (ORs) with 95% confidence intervals (CIs) was calculated with random or fixed effects model. All data analyses were analyzed by Review Manger 5.3 software. In Caucasian subgroup: Dominant model (TT + CT vs CC): OR = 1.17 (1.06, 1.29), Recessive model (TT vs CT + CC): OR = 1.25 (1.13, 1.39) and Allele frequency (T vs C): OR = 1.15 (1.08, 1.22). The present meta-analysis suggests that rs3803662 polymorphism is significantly associated with breast cancer risk in Caucasian women, and we did not find the association in Asian women.

Breast cancer, as a multifactorial disease, is the most common cancer in the world^1^. The major influences on breast cancer appear to be environmental and genetic factors^2^,^3^. Previous studies indicate that genetic influences account for about 27% of the breast cancer risk^4^. A few genes including *BRCA1*, *BRCA2*, and *ATM* have been known to be associated with the risk of breast cancer^5^. In the past 30 years, the incidence of breast cancer has been increasing rapidly in many countries. According to the data of International Agency for Research on Cancer (IARC), there are additional 1.68 million breast cancer patient in 2012, which is 25% of the women with tumor^6^.

It’s reported that genetic factors play an important role in the development of breast cancer. With the completion of the human genome project (HGP) in 2001, single nucleotide polymorphisms (SNPs) come into view as the essential factor in the development of diseases 7,8,9,10. In addition to the highly penetrant (*BRCA1, BRCA2*, and *TP53*) and moderately penetrant (*CHEK2*, *BRIP1*, *ATM* and *PALB2*) genetic variants, breast cancer has been associated with low penetrant risk (*FGFR2*, *TNRC9, MAP3K1* and *LSP1*)^11^. About 5% of breast cancer incidence is attributable to high-penetrance mutations^12^. Therefore, it’s important to evaluate the association between low penetrant and breast cancer risk.

Recent studies have identified the single nucleotide polymorphism rs3803662 associated with breast cancer risk^7^,^13^,^14^,^15^,^16^,^17^,^18^,^19^,^20^,^21^,^22^,^23^,^24^. In 2010, Rulla M Tamimi *et al*. and TV Gorodnova *et al*. found significant association in Swedish and Russian, respectively^15^,^17^. In 2011, Martha L. Slattery *et al*. found the association in Hispanic subsequently^22^. In 2014, Isabel Elematore *et al*. considered that rs3803662 was associated with breast cancer risk in Chilean^24^. In 2016, Yaning He *et al*. also find the association in Chinese Han population^23^. However, the sample size of most studies was too small. One of the aims of Meta-Analysis is leading to a higher statistical power and more robust result. Single study does not have significant result statistically sometimes. It is due to that the sample size was so small that statistical power was low. We can improve statistical power by means of combining some samples with small sample size. Therefore, we conducted a meta-analysis of the previously published studies involving SNP rs3803662 and breast cancer to get a more comprehensive result.

## Results

### Literature search

**Figure 1:**
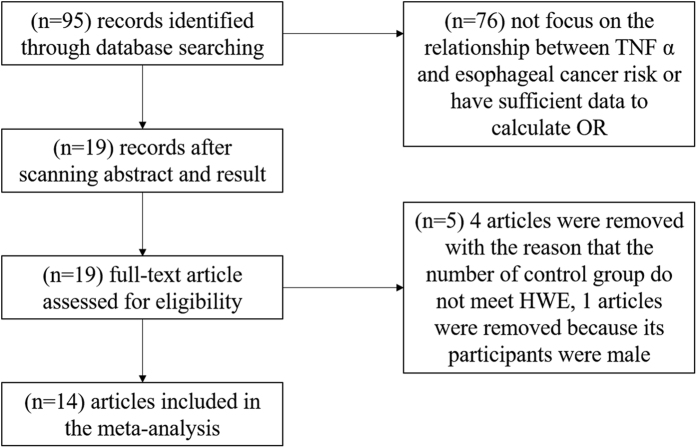
The flow diagram showing the study selection process. A total of 95 articles were identified by the search strategy. Because don’t meet the qualifications, 77 articles were removed, and 18 articles remained for further screening. Afterwards, since the control groups don’t meet the Hardy-Weinberg Equilibrium (HWE), 4 articles were excluded. Besides, 1 article was excluded due to that the study population is male, not female. Finally, 13 studies were included in the meta-analysis.

A flow diagram for the study selection process is shown in Fig. 1. A total of 95 articles were identified by the search strategy. 77 articles were removed because they don’t evaluate the association between rs3803662 and breast cancer risk or have sufficient data to calculate the ORs with 95% CIs. Afterwards, 4 articleswere excluded due to that the control group doesn’t meet the Hardy-Weinberg Equilibrium (HWE), and 1 article was excluded because that the study population is male, not female.

### Study characteristics

The primary characteristics of the 13 studies are summarized in Table 1. A total of 29405 participants with 14306 cases and 15099 controls were included in this study. The studies were divided into two subgroups according to the ethnicity of their participants: East-Asian subgroup with 4 studies and Caucasian subgroup with 8 studies, and others subgroup with 1 study.

### Association between rs3803662 polymorphism and breast cancer risk

**Figure 2:**
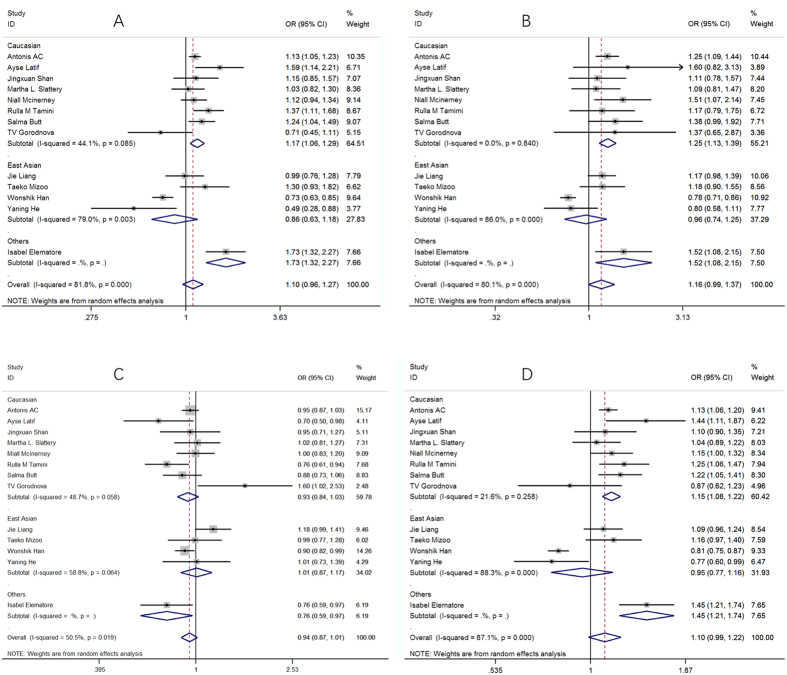
The forest plot of different model. (**A**) dominant model; (**B**) recessive model; (**C**) additive model; (**D**) allele frequency.

The forest plot concerning the association between the rs3803662 polymorphism and the risk of breast cancer is shown in Fig. 2. Figure 2A–D are forest plots of dominant model, recessive model, additive model and Allele frequency, respectively.

In Caucasian subgroup, dominant model (TT + CT vs CC): OR = 1.17 (1.06, 1.29); recessive model (TT vs CT + CC): OR = 1.25 (1.13, 1.39); additive model (TT + CC vs CT): OR = 0.93 (0.84, 1.03); allele frequency (T vs C): OR = 1.15 (1.08, 1.22).

In East Asian subgroup, dominant model (TT + CT vs CC): OR = 0.86 (0.63, 1.18); recessive model (TT vs CT + CC): OR = 0.96 (0.74, 1.25); additive model (TT+CC vs CT): OR = 1.01 (0.87, 1.17); allele frequency (T vs C): OR = 0.95 (0.77, 1.16).

### Assessment of publication bias

**Figure 3:**
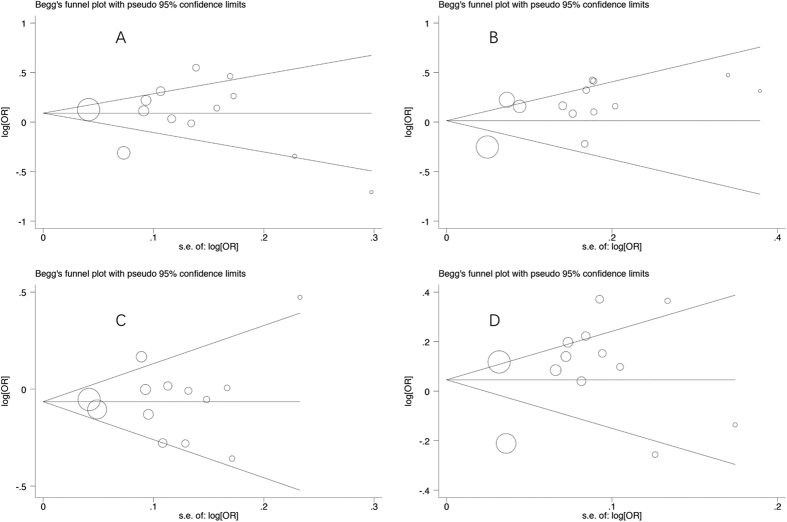
The funnel plot of different model. (**A**) dominant model; (**B**) recessive model; (**C**) additive model; (**D**) allele frequency.

Funnel plot were carried out to assess publication bias. There are many reasons leading to publication bias: low quality research with small sample, heterogeneity, and negative result. Figure 3A–D are funnel plots of dominant model, recessive model, additive model and Allele frequency, respectively. We found publication bias in Fig. 3B (recessive model). In this study, it mainly comes from few researches with small sample and heterogeneity.

## Discussion

Our meta-analyses, including 14306 cases and 15099 controls group numbers from 13 case-control studies, explored the association between the rs3803662 polymorphism and the risk of breast cancer. The result indicates that rs3803662 is significant associated with breast cancer risk in Caucasian women. And we did not find the association Asian women.

The SNP rs3803662 is significant associated with breast cancer risk in Caucasian women which is consistent with results of previous studies^15^,^16^,^17^,^18^,^19^,^20^,^21^,^22^. But in Asian women, we get inconsistent result. That can be attributed to the following reasons. First, only 4 studies with 5237 cases and 5130 controls were included in the meta-analysis according to the study selection criteria^7^,^13^,^14^,^23^. The inadequate participant results in that rs3803662 is not significantly associated with breast cancer risk in Asian women statistically. The more the studies were included in meta-analysis, the more accurate the result will be. More independent studies concentrating on the association in Asian women should be added into this analysis and we will focus on the latest research. Second, we hypothesize that genetic background varies among different ethnic populations. Furthermore, this variation leads to change in susceptibility to some cancers. The hypothesis needs a huge amount of samples to validate whether it is true or not.

The SNP rs3803662 lies 8 kb upstream of *TNRC9* which is located on the chromosome 16q12 and consists of seven exons^25^. Rs3803662 has been confined to estrogen receptor-positive tumors^26^. Though we are not familiar with the function of *TNRC9*, this gene has been found to be relevant to bone metastasis in breast cancer^27^. *TNRC9* is not only expressed in brain, but also expressed in breast with higher expression level in breast cancer compared to that in normal tissue^28^,^29^. And the minor allele of rs3803662 was associated with lower mRNA expression of *TNRC9* gene^30^. James Owain Jones *et al*. found that *TNRC9* maps to a known breast cancer susceptibility locus and hypothesized that *TNRC9* could be a candidate tumor suppressor gene in 16q^31^.

There are some limitations in this study. First, we conducted subgroup analysis according to the ethnicity of participants. Compared with Caucasian subgroup, only 4 studies with 5237 cases and 5130 controls were included in the study. Second, the variation in genetic background may have effects on the susceptibility to breast cancer. More studies focusing on the same population should be included.

In conclusion, rs3803662 polymorphism is significantly associated with breast cancer risk in Caucasian women, and we did not find the association in Asian women.

## Methods

### Search strategy

A comprehensive literature searches for suitable studies published up to 2016 was conducted in the PubMed, EMbase and Web of Science database. Studies that investigated the rs3803662 and breast cancer were included in this meta-analysis. Studies should be published as a full paper. The search was conducted using the following keywords: “rs3803662” and “breast cancer”.

### Study selection criteria

Two independent reviewers first screened the titles and abstracts to identify the relevant investigations. Then, full articles were read to include the eligible studies that met the following criteria: (1) used a case-control study design, (2) evaluated the association between rs3803662 and the risk of breast cancer, (3) provided the number of genotypes in case-controls groups for calculating ORs, (4) the control group has to satisfy HWE.

### Data extraction

For every eligible study, the following data were extracted by two independent reviewers: name of the first author, publication date, the ethnicity of study population, and the number of genotype in case-control group. In addition, the P value of HWE in control group was calculated.

### Statistical analysis

All statistical analysis was conducted by STATA version 14.0 (STATA Corporation, College Station, TX, USA). The Odds Ratios (ORs) with 95% confidence intervals (CIs) was calculated to evaluate the association between the rs3803662 polymorphism and breast cancer risk.

The heterogeneity means the variation between different researches in systematic review. It has two major types: the clinical heterogeneity and the heterogeneity of methodology. The former comes from the difference of participants, interventions and outcome among studies. The latter derives from distinction of experimental design. We use *Q* and *I*^*2*^ statistics to assess the heterogeneity. If *I*^2^ < 50% or the *P*-value of heterogeneity >0.10, we use fixed effects model. Otherwise, the random effects model was selected.

The Begg’s rank correlation test and Egger’s linear regression test were used to assess the publication bias. The genetic models we use here include dominant model, recessive mode, additive model and allele frequency^32^,^33^.

## Additional Information

**How to cite this article**: Yang, Y. *et al*. Association of single nucleotide polymorphism rs3803662 with the risk of breast cancer. *Sci. Rep.* **6**, 29008; doi: 10.1038/srep29008 (2016).

## Figures and Tables

**Table 1:** The primary characteristics of the thirteen studies.

Author (Publication Date)	Country	Case			Control			*P* value of HWE
		CC(%)	CT(%)	TT(%)	CC(%)	CT(%)	TT(%)	
Jie Liang (2010)	Chinese	126 (12.3)	413 (40.3)	486 (47.4)	127 (12.1)	464 (44.4)	455 (43.5)	0.603467
Taeko Mizoo (2013)	Japanese	74 (15.9)	230 (49.6)	160 (34.5)	91 (19.8)	227 (49.3)	142 (30.9)	0.986928
Wonshik Han (2011)	South Korean	516 (14.8)	1617 (46.3)	1361 (38.9)	369 (11.2)	1435 (43.7)	1481 (45.1)	0.3174
Yaning He (2016)	Chinese	30 (11.8)	115 (45.3)	109 (42.9)	21 (6.2)	154 (45.4)	164 (48.4)	0.052716
Isabel Elematore (2014)	Chilean	100 (28.8)	185 (53.3)	62 (17.9)	330 (41.2)	371 (46.3)	100 (12.5)	0.786243
Antonis AC (2008)	Caucasian	2422 (47.5)	2173 (42.7)	497 (9.8)	2244 (50.3)	1831 (41.1)	382 (8.6)	0.756163
Ayse Latif (2009)	England	106 (46.7)	103 (45.4)	18 (7.9)	217 (58.2)	137 (36.7)	19 (5.1)	0.659871
Jingxuan Shan (2012)	Tunisian	114 (31.1)	169 (46.0)	84 (22.9)	126 (34.1)	165 (44.7)	78 (21.2)	0.082818
Martha L. Slattery (2011)	Hispanic	209 (37.1)	260 (46.1)	95 (16.8)	270 (37.8)	332 (46.5)	112 (15.7)	0.554053
Niall Mcinerney (2009)	Irish	486 (51.2)	382 (40.2)	82 (8.6)	532 (53.9)	396 (40.1)	58 (6.0)	0.160713
Rulla M Tamimi (2010)	Swedish	333 (48.5)	300 (43.6)	54 (7.9)	415 (56.2)	273 (36.9)	50 (6.9)	0.575927
Salma Butt (2012)	Swedish	353 (50.8)	278 (40.0)	64 (9.2)	780 (56.2)	512 (36.9)	95 (6.9)	0.380434
TV Gorodnova (2010)	Russian	74 (52.8)	50 (35.8)	16 (11.7)	77 (44.2)	82 (47.2)	15 (8.6)	0.293717

A total of 29405 participants with 14306 case group numbers and 15099 control group numbers were included in this study. It includes name of the first author, publication date, the country of study population, the number of genotype in case-control group and theP value of HWE. Besides, the P value of HWE belongs to the control group and it help us find whether the control group meets the selection criteria(P > 0.05).
